# Effects of green tea dust on the biochemical parameters, antioxidant capacity, and intestinal microbiota composition in goose

**DOI:** 10.3389/fvets.2025.1694350

**Published:** 2025-12-04

**Authors:** Zhuoya Gu, Wenwu Xu, Yong Tian, Lizhi Lu, Guohong Chen

**Affiliations:** 1 Jiangsu Key Laboratory for Animal Genetic, Breeding and Molecular Design, Yangzhou University, Yangzhou, China; 2 Institute of Animal Husbandry and Veterinary Science, Zhejiang Academy of Agricultural Sciences, Hangzhou, China

**Keywords:** tea dust, antioxidant capacity, intestinal absorption capacity, gut microbiota, goose

## Abstract

**Introduction:**

Green tea dust (GTD), a by-product of tea processing, exhibits promising potential as a functional feed additive owing to its rich protein profile and bioactive compounds.

**Methods:**

This study evaluated the impact of GTD inclusion on biochemical parameters, oxidative stress markers, intestinal morphology, and cecal microbiota in Zhedong White geese. A cohort of 120 21-day-old male geese was randomly allocated to four dietary regimens: a basal diet (CTRL) and basal diets supplemented with 10% (LGTD), 15% (MGTD), or 20% ET (HGTD). The experimental period lasted seven weeks.

**Results:**

Results indicated that GTD supplementation exerted no significant influence on plasma lipid metabolism. However, graded GTD doses markedly elevated systemic antioxidant activity, as evidenced by improved plasma antioxidant indices. Morphometric analysis revealed enhanced intestinal absorptive function, characterized by increased villus height (VH), reduced crypt depth (CD), and elevated VH/CD ratios in the duodenum. Furthermore, GTD supplementation modulated cecal microbial communities, promoting a favorable microbiota profile.

**Conclusion:**

These findings underscore the utility of GTD as a dietary intervention to augment intestinal health and oxidative status in geese, providing empirical support for its broader adoption in poultry nutrition.

## 1 Introduction

Modern poultry production systems grapple with the dual challenges of sustaining growth efficiency and mitigating health risks under high-density rearing conditions. Of particular concern are oxidative stress and intestinal dysfunction, which compromise nutrient utilization, product quality, and sustainability (1). In response, the exploration of functional feed additives—particularly those derived from natural by—products—has gained traction as a strategy to enhance physiological resilience. Bioactive compounds, such as polyphenols and polysaccharides, are increasingly recognized for their dual capacity to modulate redox homeostasis and gastrointestinal health, offering a viable alternative to conventional growth promoters (2).

Green tea (*Camellia sinensis*) is a rich source of bioactive compounds, including polyphenols, polysaccharides, amino acids, and vitamins, which contribute to its well-documented pharmacological properties (3, 4). As a by-product of tea processing, green tea dust (GTD) retains many of these functional components but at a significantly reduced cost. Numerous studies have indicated the efficacy of green tea dust in anti-inflammation and antioxidant activity, due to its ingredients rich in tea polyphenols and tannins (5, 6). While studies in ruminants, such as sheep, have shown that GTD supplementation improves performance without compromising nutrient digestibility (7), its potential as a dietary supplement in poultry remains unexplored.

The intestinal tract is a critical site for nutrient assimilation, immune modulation, and host-microbiome interactions, with profound implications for overall animal health (8–11). Emerging evidence suggests that dietary interventions can modulate gut microbial composition and enhance systemic antioxidant responses (12–14). For instance, polysaccharide-based feed additives in poultry have been shown to promote microbial diversity, particularly enriching *Firmicutes* and *Verrucomicrobiota*, leading to improved growth performance, redox balance, and intestinal morphology (15). Similarly, supplementation with bioactive compounds such as tea tree oil has demonstrated efficacy in enhancing growth metrics and immune function through selective modulation of key microbial taxa (e.g., *Clostridiaceae_1*) (16).

The digestive physiology of geese, characterized by a highly developed cecal fermentation system, presents unique opportunities for utilizing fibrous feed ingredients to modulate microbial ecology and enhance oxidative resilience (3). As an economically significant indigenous breed in China, the Zhedong white goose serves as an ideal model to investigate the functional effects of unconventional dietary components. Thus, this study aimed to test the hypothesis that green tea dust supplementation can play role in antioxidant status and gut health as an additive in the diet of Zhedong White geese.

## 2 Materials and methods

### 2.1 Experimental design and sample collection

Male Zhedong White geese and green tea dust (a discarded waste material after industrial production of green tea beverages) were provided from the Zhejiang White Goose Research Institute and Tea Research Institute from Chinese Academy of Agricultural Sciences, respectively. One hundred and twenty 21-day-old healthy male Zhedong White geese were randomly divided into four groups: CTRL (the basal diet), LGTD (the basal diet with 10% tea dust), MGTD (the basal diet with 15% tea dust), and HGTD (the basal diet with 20% tea dust). The experimental period lasted seven weeks. At trial termination, six geese were randomly selected for sample collection and analysis. Dietary formulations and nutritional composition are detailed in Table 1.

**Table 1:** The composition and nutrient levels of the experimental diets.

Items	1 ~ 42 d
Ingredient (%)	
Corn	14.30
Wheat	35.00
Soybean meal	2.20
Corn protein power	3.40
Rice bran	26.30
Wheat bran	18.00
Sodium chloride	0.40
Premix^1^	0.40
Total	100
Nutrient composition, calculated	
Metabolizable energy (MJ/kg)	11.01
Crude protein (%)	14.78
Crude fat (%)	4.82
Calcium (%)	0.88
Total phosphorus (%)	0.62
Lysine (%)	0.90
Methionine (%)	0.50

^1^Premix provided per kilogram of diet: vitamin A, 15, 000 IU; vitamin D5, 300 IU; vitamin E, 100 mg; vitamin B1, 2 mg; vitamin B2, 1, 200 mg; vitamin B6, 3.85 mg; vitamin B12, 3.85 mg; Cu (as CuSO4·5H2O), 20 mg; Fe (as FeSO4·7H2O), 70 mg; Zn (as ZnSO4), 100 mg; Mn (as MnSO4), 80 mg; I (as Ca(IO3)2), 3 mg; and Se (as NaSeO3·5H2O), 0.50 mg.

Following the 7-week experimental period, blood was collected via subcutaneous venipuncture and processed by centrifugation (3,000 × *g*, 10 min, 4 °C) to isolate plasma, which was subsequently stored at −80 °C. Geese were humanely euthanized via intravenous sodium pentobarbital administration (200 mg/kg BW). Tissue specimens (liver, duodenum, and cecum content) were excised, flash-frozen in liquid nitrogen, and preserved at −80 °C for subsequent nutritional profiling and microbial genomic analysis.

### 2.2 Biochemical analysis

Liver total cholesterol (TC), triglycerides (TG), and non-esterified fatty acids (NEFA) were measured using commercially available kits (Jiancheng Biotechnology Inc., Nanjing, China) following the manufacturers’ instructions.

### 2.3 Oxidative stress biomarkers

Plasma oxidative status was assessed by measuring the total antioxidant capacity (T-AOC) and the activities of antioxidant enzymes (CAT, SOD, GSH-PX) using commercial assay kits (Solarbio, Beijing, China), with lipid peroxidation evaluated through malondialdehyde (MDA) quantification according the manufacturers’ instructions (Solarbio).

### 2.4 Histological observation

Duodenal samples were fixed in 4% paraformaldehyde and processed for histological analysis. Following dehydration in xylene (15–20 min) and paraffin embedding at 60 °C, 5-μm sections were prepared and stained with hematoxylin–eosin. Morphometric evaluation was performed by optical microscopy, with five serial sections analyzed per sample. From each section, six representative fields containing intact villi were selected for measurement. The sections were analyzed under an Olympus light microscope (Tokyo, Japan). Villus height was determined from the longest villus in each field, with corresponding crypt depth measured simultaneously. Mean values were calculated from all measurements for statistical analysis. All methods and detection were conducted by servicebio (Wuhan, China).

### 2.5 Microbial community analysis

Genomic DNA from cecal samples was isolated using a commercial extraction kit (Omega, Norcross, GA). The V3-V4 hypervariable regions of bacterial 16S rRNA genes were amplified using universal primers (341F: ACTCCTACGGGAGGCAGCA; 806R: GGACTACHVGGGTWTCTAAT). Amplicon libraries were prepared and sequenced on an Illumina MiSeq platform (2 × 300 bp) by Personalbio Technology (Shanghai). Sequence processing was conducted in QIIME 2, where amplicon sequence variants (ASVs) were generated through DADA2 denoising. Microbial diversity was assessed using *α*-diversity indices (Shannon, Chao1) and *β*-diversity metrics visualized via principal coordinate analysis (PCoA). Differential abundance of taxa between groups was determined using linear discriminant analysis effect size (LEfSe) with an LDA score threshold >2.0.

### 2.6 Statistical analysis

Data are presented as mean ± SD. Differences between groups were assessed by one-way ANOVA (SPSS 25.0), with statistical significance set at *p* < 0.05. Graphical representations were generated using GraphPad Prism 8.0.

## 3 Result

### 3.1 Lipid parameters

To determine whether dietary addition of tea dust could affect the fat deposition in body, the contents of TC, TG, and NEFA were analyzed. As shown in Table 2, dietary supplementation with different dose of tea dust did not alter the levels of plasma TC, TG, and NEFA (*p* > 0.05).

**Table 2:** Effect of tea dust supplementation on plasma lipid levels in Zhedong White geese.

Item	CTRL	LET	MET	HET
TC (mmol/L)	1.705 ± 0.373	1.879 ± 0.307	1.773 ± 0.108	1.724 ± 0.114
TG (mmol/L)	0.441 ± 0.100	0.299 ± 0.007	0.298 ± 0.025	0.375 ± 0.052
NEFA (mmol/L)	0.334 ± 0.058	0.404 ± 0.080	0.444 ± 0.072	0.392 ± 0.057

TC, total cholesterol; TG, triglyceride; NEFA, non-esterified fatty acids. *n* = 6 for each group.

### 3.2 Antioxidant capacity

**Figure 1:**
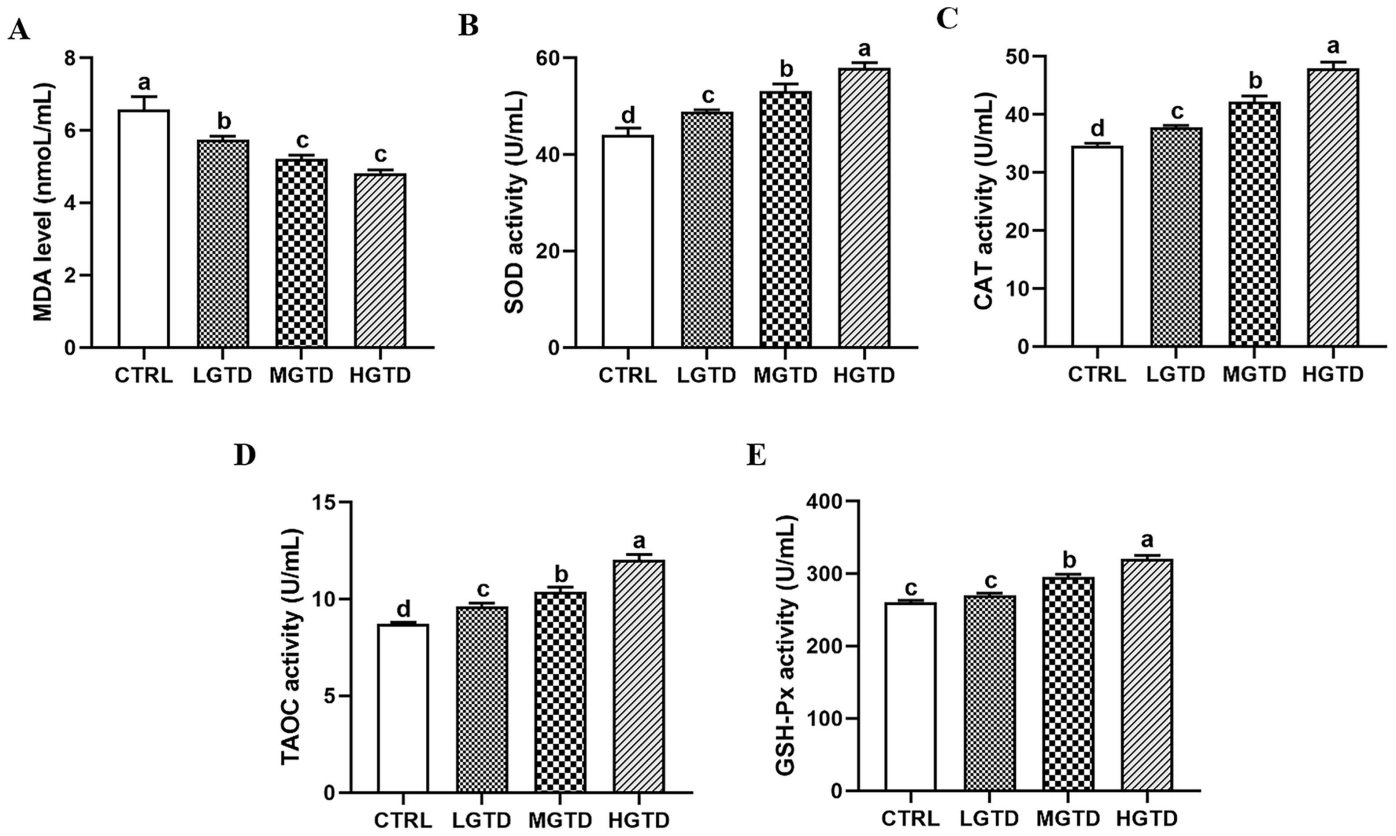
Effect of tea dust supplementation on plasma antioxidant capacity in Zhedong White geese. **(A)** MDA level. **(B)** SOD activity. **(C)** CAT activity. **(D)** TAOC activity. **(E)** GSH-PX activity. *n* = 6 for each group. ^a–d^Means with different letters are significantly different (*p* < 0.05).

Plasma oxidative status parameters responded dose-dependently to tea dust supplementation (Figure 1). The tea dust supplementation groups exhibited significantly lower MDA levels than CTRL group (*p* < 0.05; Figure 1A) and the activities of antioxidant enzymes (SOD, CAT, T-AOC, GSH-PX) were significantly elevated compared to controls (*p* < 0.05; Figures 1B–D).

### 3.3 Intestinal morphology analysis

**Figure 2:**
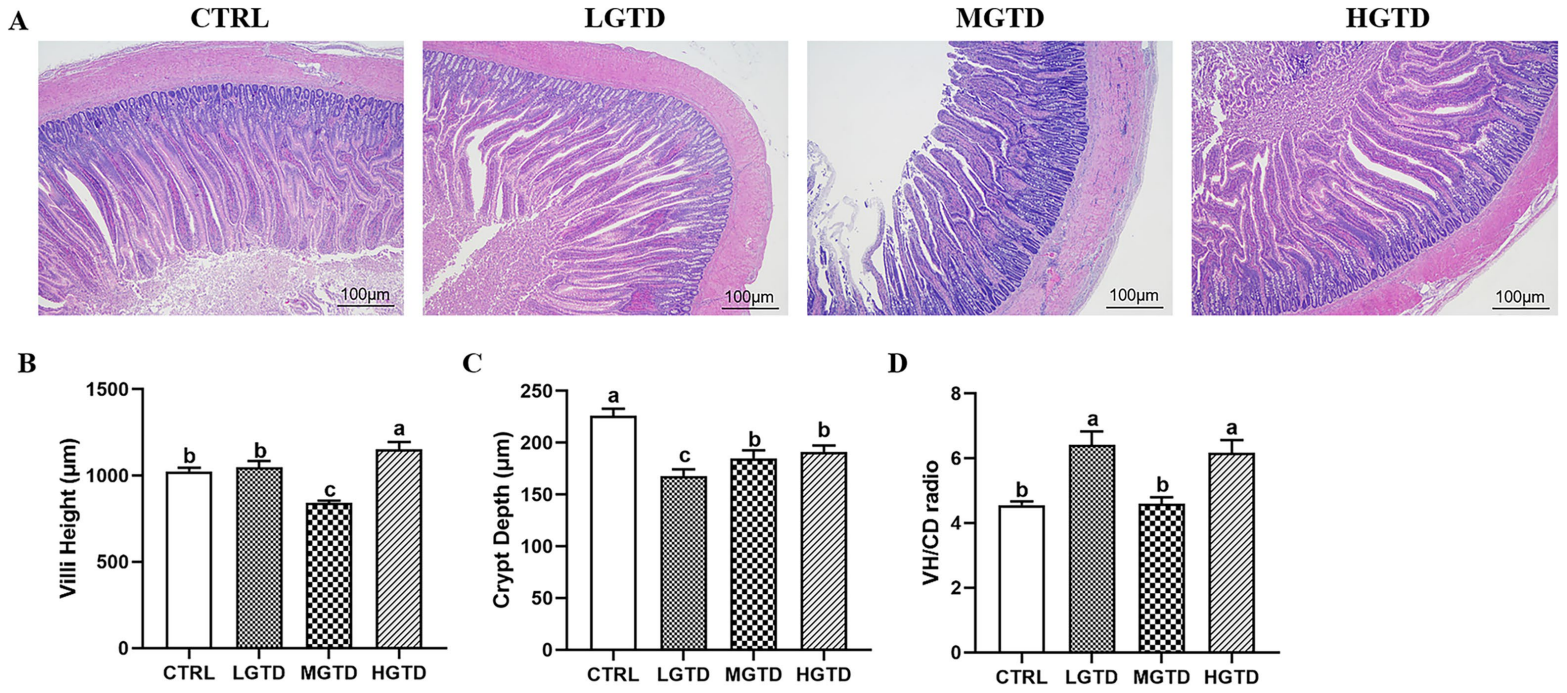
Effect of tea dust supplementation on the duodenal morphology analysis in Zhedong White geese. **(A)** HE staining. **(B)** Villi height (VH). **(C)** Crypt depth (CD). **(D)** VH/CD radio. Magnification of 200X was used (Bar = 100 μm). *n* = 6 for each group. ^a–c^Means with different letters are significantly different (*p* < 0.05).

To determine the effects of tea dust on the intestinal absorption capacity, villi height (VH), crypt depth (CD) and VH/CD radio of the duodenum were assessed. As shown in Figure 2, duodenal histopathological analysis showed that the VH was significantly longer in the LGTD and HGTD group than that in the CTRL and HET group (*p* < 0.05). The CD was significantly smaller in the LGTD, MGTD and HGTD group than that in the CTRL group (*p* < 0.05). The VH/CD, which indicated the wall thickness of duodenum, was significantly greater in the LGTD and HGTD group than that in the CTRL and HGTD (*p* < 0.05). These findings indicated that tea dust could affect the morphology to improve the absorption capacity of the intestine.

### 3.4 Gut microbiota compositions

**Figure 3:**
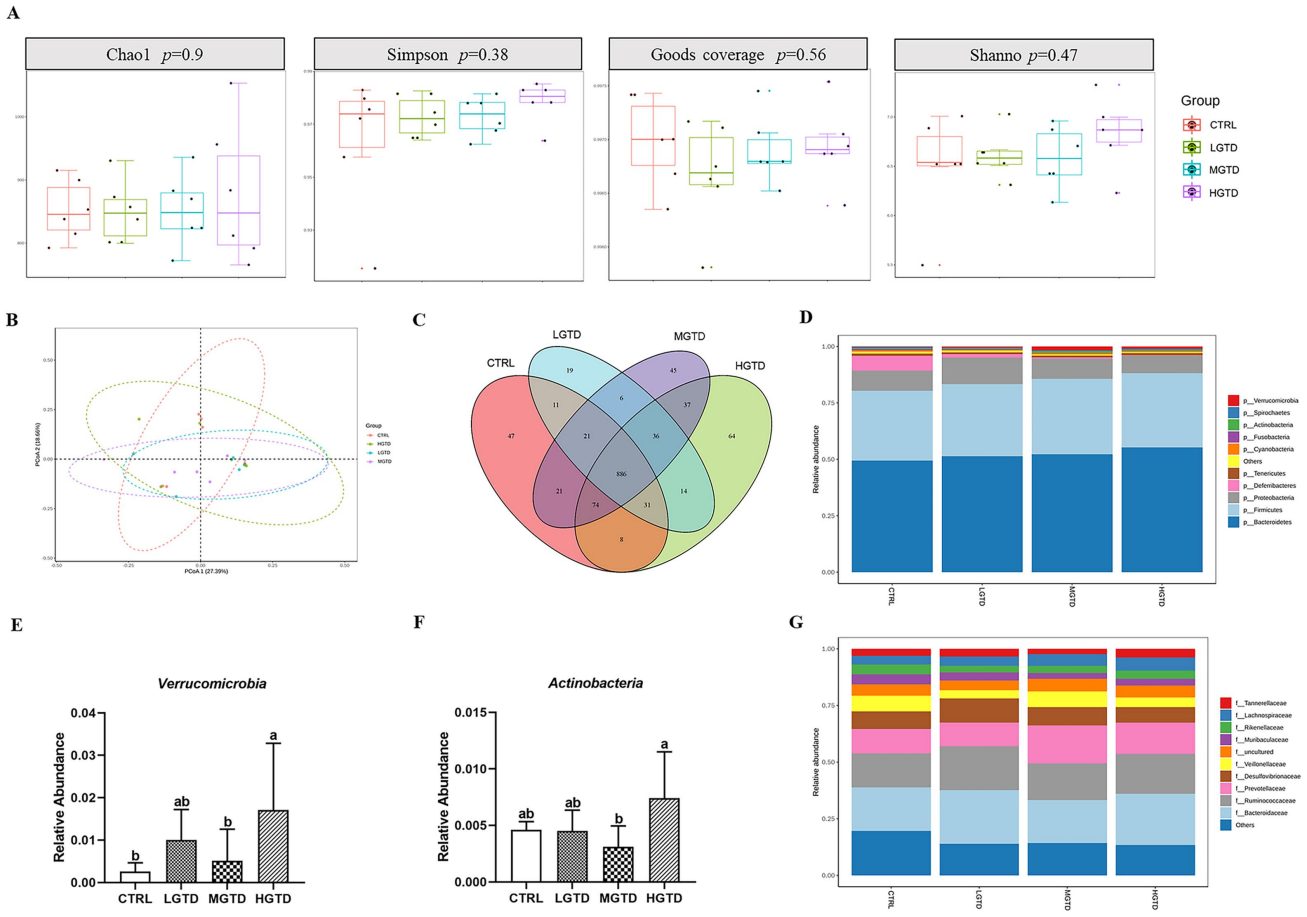
Effects of tea dust supplementation on the gut microbiota compositions in geese. **(A)** Variations in *α* diversity. **(B)** PCoA analysis. **(C)** Venn diagram. **(D)** Microbiota composition analysis at the phylum level. **(E,F)** The relative abundance of *Verrucomicrobia* and *Actinobacteria*. **(G)** Microbiota composition analysis at the family level. *n* = 6 for each group.

**Figure 4:**
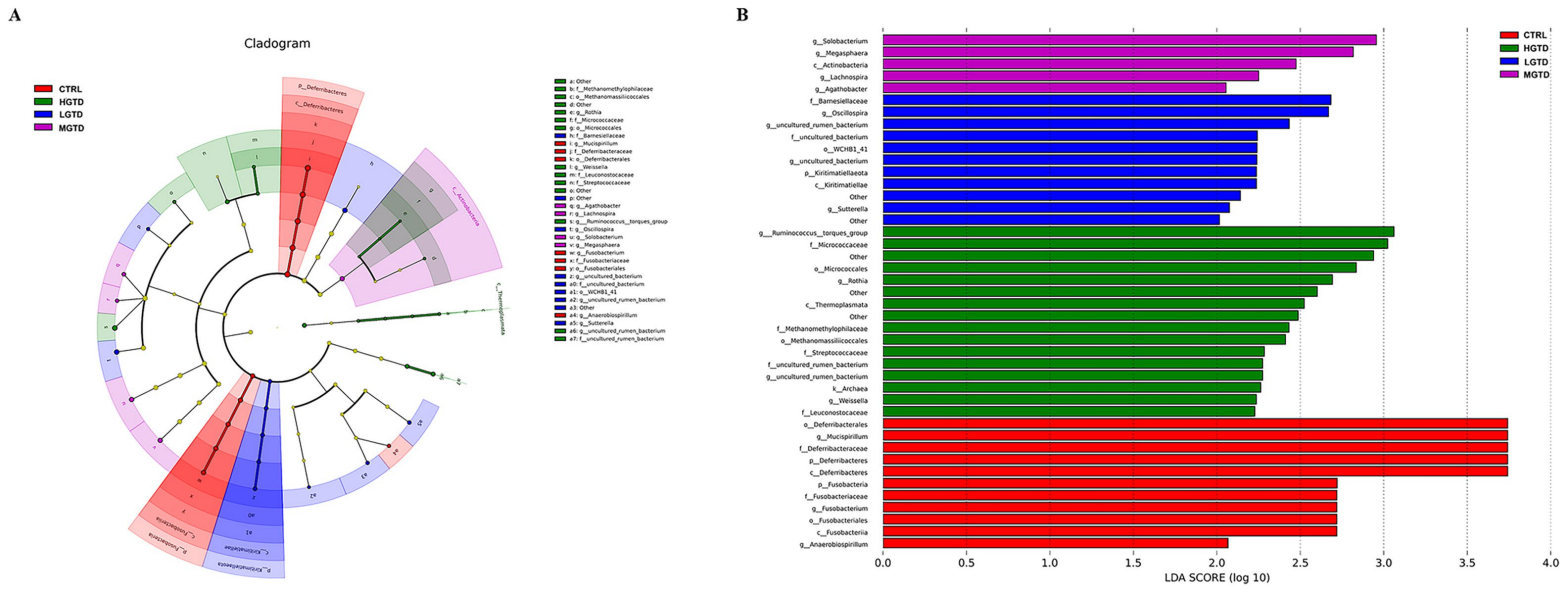
Analysis of the diversity of microbial communities with LEfSe analysis. **(A)** Cladogram. **(B)** LDA score.

To assess gut microbiota modulation by tea dust supplementation, we analyzed cecal microbiomes via 16S rRNA sequencing. The *α* diversity (represented by the Chao1, Simpson, Goods coverage, and Shannon indices) of the intestinal flora remained unchanged across groups (*p* > 0.05, Figure 3A). *β*-diversity analysis revealed overlapping microbial communities between CTRL and supplemented groups (Figure 3B). A Venn diagram showed that 886 amplicon sequence variants (ASVs) of the microbiota were obtained among the CTRL, LGTD, MGTD and HGTD groups (47, 19, 45, and 64 unique ASVs, respectively) (Figure 3C). As shown in Figure 3D, *Bacteroidetes*, *Firmicutes* and *Proteobacteria* were the most abundant phyla. Compared to the CTRL group, the relative abundance of *Verrucomicrobia* was significantly increased in HGTD group and the relative abundance of *Actinobacteria* was significantly decreased in MGTD group (Figures 3E,F). Furthermore, *Bacteroidaceae*, *Ruminococcaceae*, and *Prevotellaceae* were the dominant family (Figure 3G). LEfSe analysis showed that the genera *Anaerobiospirillum*, *Fusobacterium*, and *Mucispirillum* in the CTRL group, *Weissella*, *Rothia*, and *Ruminococcus torques_group* in the LGTD group, *Sutterella*, and *Oscillospira* in the MGTD group, and *Lachnospira*, *Agathobacter*, *Solobacterium*, and *Megasphaera* in the HGTD group were the predominant bacterial strains (Figure 4).

## 4 Discussion

Green tea (*Camellia sinensis*), a non-fermented tea variety, has been extensively studied for its notable health-promoting properties (17, 18). Compared to other tea types, green tea exhibits particularly significant bioactive effects, with reported benefits in managing obesity (19, 20), modulating gut microbiota (21), reducing cancer risk (22, 23), improving cardiovascular health (24, 25), alleviating osteoarthritis symptoms (26), and mitigating metabolic disorders such as hypercholesterolemia and hyperglycemia (27). Our previous study demonstrated that green tea dust supplementation improved growth and slaughter performance in geese (28). In this study, we further confirmed green tea dust could enhance antioxidant capacity, intestinal microbiota composition in geese, while not altered lipid metabolism.

Several serum lipid measurements, such as TC, TG, and NEFA, are indicative of lipid metabolism status in general (29). TG converts to glycerol and free fatty acids in liver (30). Non-esterified fatty acids (NEFAs), primarily derived from adipose tissue lipolysis, serve as a key contributor to the hepatic triglyceride (TG) pool. Elevated circulating NEFA levels are associated with the development of insulin resistance in skeletal muscle and liver, as well as the onset of dyslipidemia (31). In this study, TG, TC and NEFA concentration in serum of geese showed no significance with dietary tea dust supplementation, suggesting that tea dust has little effect in lipid metabolism.

Redox homeostasis is essential for maintaining normal metabolic and physiological functions. Disruption of this equilibrium can lead to metabolic dysregulation and uncontrolled free radical reactions, contributing to oxidative stress (32, 33). Endogenous antioxidant enzymes, including superoxide dismutase (SOD), catalase (CAT), and glutathione peroxidase (GSH-Px), constitute a primary defense system against oxidative damage (34, 35). SOD serves as the initial scavenger of reactive oxygen species (ROS), catalyzing the dismutation of superoxide anions (O₂^−^) into hydrogen peroxide (H₂O₂) and oxygen (O₂) (36). Subsequently, CAT facilitates the decomposition of H₂O₂ into water and molecular oxygen (37), while GSH-Px further eliminates H₂O₂ and detoxifies lipid peroxides, thereby protecting cellular integrity (38). In the present study, dietary supplementation with tea dust significantly enhanced plasma antioxidant capacity, as evidenced by elevated SOD, CAT, GSH-Px, and total antioxidant capacity (T-AOC) activities, alongside a marked reduction in malondialdehyde (MDA) levels. These findings align with previous reports demonstrating that fermented tea residue feed boosts SOD and GSH-Px activities while lowering serum MDA in laying hens (39). Similarly, dietary tea polyphenols have been shown to upregulate GSH-Px and T-AOC while suppressing MDA accumulation in poultry, further supporting the antioxidative potential of tea-derived compounds (40). This indicates that dietary with tea dust can reduce oxidative stress in geese.

The intestine is a key organ that helps digest and absorb nutrients. It also acts as a barrier to protect the body and is involved in cell communication (41, 42). Two important measurements of intestinal health are villus height (VH) and crypt depth (CD) (43). In the present study, dietary inclusion of 20% tea dust significantly reduced CD while increasing VH and the villus height-to-crypt depth ratio (V/C) compared to the control group. These morphological improvements suggest that tea dust supplementation enhances intestinal villus development, potentially optimizing nutrient assimilation and gut health (44).

The avian gut microbiota predominantly colonizes the cecal region, playing pivotal roles in host metabolism, immune modulation, and nutrient utilization (45, 46). Our findings revealed that while dietary tea dust supplementation did not significantly alter the relative abundance of dominant phyla *Bacteroidetes* and *Firmicutes*, it notably increased *Verrucomicrobia* and reduced *Actinobacteria* populations in the geese cecum. These microbial shifts suggest tea dust may selectively modulate specific bacterial taxa without disrupting the overall phylum-level composition. A previous study demonstrated that *Verrucomicrobia* usually represents a minor population of intestinal microbiota in response to dietary shifts in mice (47). Furthermore, *Actinobacteria* play a significant role in fiber degradation, particularly in metabolizing plant-based carbohydrates such as starch, inulin, and arabinoxylan (48). Notably, studies have shown that dietary CR or FCR supplementation leads to elevated Actinobacteria populations specifically in the ileal region (49). LEfSe analysis further validated the selective modulation of cecal microbiota by tea dust, suggesting that tea dust supplementation may enhance intestinal health through targeted microbial community restructuring. The above data indicated that the antioxidant capacity conferred by green tea dust supplementation is associated with the modulation of gut microbiota. Supporting this, tea polyphenol-induced amelioration of gut microbiota showed a strong correlation in enhancing antioxidant activity in mice (50). This evidence underpins the further exploration of the prebiotic potential inherent to green tea dust.

## 5 Conclusion

Collectively, our findings demonstrate that 20% tea dust inclusion in the diet enhances antioxidant capacity, intestinal function, and microbial ecology in Zhedong White geese. These physiological improvements support the practical application of tea dust supplementation as a nutritional strategy to optimize goose production performance.

## Data Availability

The datasets presented in this study can be found in online repositories. The names of the repository/repositories and accession number(s) can be found at: https://ngdc.cncb.ac.cn/gsa/index.jsp, CRA032681.
